# Clonal haematopoiesis in chronic lymphocytic leukaemia: Biology, inflammaging and clinical implications in the era of targeted therapy

**DOI:** 10.1002/ctm2.70633

**Published:** 2026-03-07

**Authors:** Enrica Antonia Martino, Santino Caserta, Mamdouh Skafi, Maria Eugenia Alvaro, Antonella Bruzzese, Nicola Amodio, Eugenio Lucia, Virginia Olivito, Caterina Labanca, Francesco Mendicino, Ernesto Vigna, Fortunato Morabito, Massimo Gentile

**Affiliations:** ^1^ Unit of Hematology Department of Onco‐Hematology AO of Cosenza Cosenza Italy; ^2^ Department of Emergency and Internal Medicine Saint Joseph Hospital East Jerusalem Palestine; ^3^ Department of Experimental and Clinical Medicine University of Catanzaro Catanzaro Italy; ^4^ AIL Sezione di Cosenza Cosenza Italy; ^5^ Department of Pharmacy Health and Nutritional Science University of Calabria Rende Italy

**Keywords:** chronic lymphocytic leukaemia, clonal haematopoiesis, inflammaging, prognostic biomarkers, targeted and preventive strategies, therapy‐driven clonal evolution

## Abstract

**Background:**

Clonal haematopoiesis (CH) is an age‐related condition increasingly recognised for its relevance in haematologic malignancies. In chronic lymphocytic leukaemia (CLL), its prevalence and clinical implications are gaining attention, particularly in the context of prolonged patient survival and the widespread adoption of targeted therapies. A comprehensive understanding of the biological and clinical significance of CH in CLL is therefore essential.

**Methods:**

This review synthesises current evidence on the biological basis, epidemiology and clinical impact of CH in CLL. Data from prospective clinical trials, real‐world cohorts and translational studies were analysed to explore the associations between CH, genomic instability, immune dysregulation and inflammaging. Particular attention was given to the interaction between CH and contemporary therapeutic strategies, including Bruton tyrosine kinase (BTK) inhibitors and BCL2 inhibitors, and their potential influence on long‐term outcomes.

**Results:**

Available evidence indicates that CH is relatively frequent in patients with CLL and may contribute to disease biology through mechanisms involving genomic instability, chronic inflammation and immune system alterations. Emerging data suggest that CH can influence prognosis, treatment‐related toxicities and cardiovascular risk, as well as predispose to therapy‐related myeloid neoplasms. The interplay between CH and targeted agents may further modulate long‐term outcomes, although the impact of CH on Richter transformation remains incompletely defined.

**Conclusions:**

CH represents a clinically relevant factor in the management of CLL in the era of targeted therapies. Its detection may have important implications for risk stratification, toxicity monitoring and survivorship care. Further prospective studies are needed to clarify its prognostic value and to integrate CH assessment into routine clinical practice and personalised treatment algorithms.

**Key points:**

- Clonal haematopoiesis (CH) is common in patients with chronic lymphocytic leukaemia (CLL) and reflects age‐related genomic and inflammatory remodeling of haematopoiesis.
- CH may influence prognosis, treatment‐related toxicities, cardiovascular risk, and the development of therapy‐related myeloid neoplasms.
- Targeted therapies, including BTK and BCL2 inhibitors, interact differently with CH compared with chemoimmunotherapy, potentially mitigating some adverse effects.
- Integrating CH assessment into CLL management may improve risk stratification and long‐term survivorship strategies.

## Graphical abstract

In an ageing and inflamed haematopoietic ecosystem, clonal haematopoiesis and chronic lymphocytic leukaemia may originate from shared or parallel stem cell clones. This interaction modulates the effects of targeted therapies and contributes to cytopenias, cardiovascular toxicity, therapy‐related myeloid neoplasms and Richter transformation.

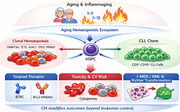

## 1 INTRODUCTION

Chronic lymphocytic leukaemia (CLL) is characterised by the accumulation of mature CD5^+^CD19^+^CD23^+^ B lymphocytes and displays a highly heterogeneous clinical course, ranging from indolent disease to aggressive, treatment‐refractory leukaemia.^1^, ^2^, ^3^ This variability reflects interactions between inherited susceptibility, acquired genetic and epigenetic lesions, microenvironmental signals and immune dysregulation.

Early prognostic models relied on recurrent cytogenetic abnormalities, including deletion 13q14, trisomy 12, deletion 11q22–23 (ATM) and deletion 17p13 (TP53). Isolated del(13q) is generally associated with indolent disease, whereas del(11q) and del(17p) predict aggressive behaviour and resistance to chemoimmunotherapy.^4^, ^5^ Next‐generation sequencing (NGS) further refined risk stratification by identifying recurrent somatic mutations—such as NOTCH1, SF3B1, ATM, TP53, BIRC3, MYD88, POT1 and NFKBIE—accounting for inter‐individual variability in disease course and treatment response.^6^, ^7^, ^8^, ^9^ Longitudinal studies demonstrated that CLL is genetically dynamic, with therapy‐driven subclonal selection underlying progression and relapse.^10^, ^11^, ^12^

In parallel, population‐based sequencing revealed that haematopoietic stem and progenitor cells (HSPCs) in healthy individuals frequently acquire somatic mutations, most commonly in DNMT3A, TET2 and ASXL1, defining clonal haematopoiesis (CH) when clonal expansion occurs without overt malignancy. The term clonal haematopoiesis of indeterminate potential (CHIP) applies when the variant allele frequency (VAF) is ≥2% in the absence of cytopenia or myeloid malignancy.^13^, ^14^, ^15^, ^16^, ^17^ CHIP prevalence increases with age, affecting 10%–20% of individuals over 70 years, and is associated with an increased relative risk of myeloid neoplasms, although the absolute risk remains low.^14^, ^16^, ^18^, ^19^

CHIP is now recognised as a systemic, age‐related condition associated with increased cardiovascular morbidity and mortality.^20^, ^21^, ^22^, ^23^ Mechanistically, TET2‐ or DNMT3A‐deficient macrophages acquire a pro‐inflammatory phenotype that sustains chronic inflammation and accelerates atherosclerosis.^24^, ^25^, ^26^, ^27^ CLL and CH are therefore increasingly viewed as biologically connected. Both are age associated, may originate from HSPCs, and share recurrent lesions such as TP53 and SF3B1 that can arise at the hematopoietic stem cells (HSC) level and propagate across lineages.^28^, ^29^ Pre‐diagnostic sequencing has identified CLL‐like clones years before clinical diagnosis, and monoclonal B‐cell lymphocytosis (MBL) frequently coexists with myeloid CH, supporting overlapping pre‐leukaemic states.^29^, ^30^, ^31^, ^32^, ^33^, ^34^

CLL represents an ideal model to study how CH influences the course of an established malignancy. Historical chemoimmunotherapy regimens imposed substantial genotoxic stress and were associated with therapy‐related myeloid neoplasms (therapy‐related myelodysplastic syndrome/acute myeloid leukaemia [t‐MDS/AML]).^35^, ^36^ Many such cases originate from pre‐existing CH clones—particularly those harbouring TP53 or PPM1D mutations—selected by cytotoxic therapy, rather than caused by it, reframing treatment as a selective force.^37^, ^38^, ^39^, ^40^, ^41^

Targeted agents, including Bruton tyrosine kinase inhibitors (BTKis) and venetoclax, have shifted CLL management towards chemo‐free strategies.^42^, ^43^, ^44^, ^45^ While reducing genotoxic exposure, these therapies exert selective pressure on CH. BTK inhibition affects immune signalling and cardiovascular risk, whereas venetoclax selects for alterations in apoptotic pathways; notably, BAX‐mutated CH has been reported during venetoclax therapy and may arise predominantly in non‐B‐cell lineages rather than driving CLL resistance.^46^

Until recently, the prevalence and clinical relevance of CH in prospective CLL cohorts were incompletely defined. Earlier studies reported CHIP in 10%–24% of patients.^41^, ^47^, ^48^, ^49^ This was revised by Al‐Sawaf et al., who analysed patients enrolled in the German CLL Study Group phase III CLL12 (ibrutinib vs. placebo) and CLL14 (venetoclax–obinutuzumab [Ven‐Obi] vs. chlorambucil–obinutuzumab [Clb‐Obi]) trials.^50^ CH was detected in ∼58% of patients (∼35% with CHIP), with mutations dominated by DNMT3A, TET2, TP53 and ASXL1. Gene‐ and regimen‐specific selection patterns were observed, and the prognostic impact of CH proved context dependent—adverse in untreated or chlorambucil‐treated patients, but largely neutral with ibrutinib or Ven‐Obi.

Additional cohorts beyond CLL12/CLL14, including smaller single‐centre or retrospective studies from the United States, Italy and UK, similarly report prevalent CH in CLL patients, confirming that this phenomenon is not restricted to German trial populations.^51^, ^52^

Collectively, these findings establish CH as a central component of the biological and clinical landscape of CLL, with implications for risk stratification, treatment selection, toxicity, cardiovascular risk and long‐term survivorship.

**FIGURE 1:**
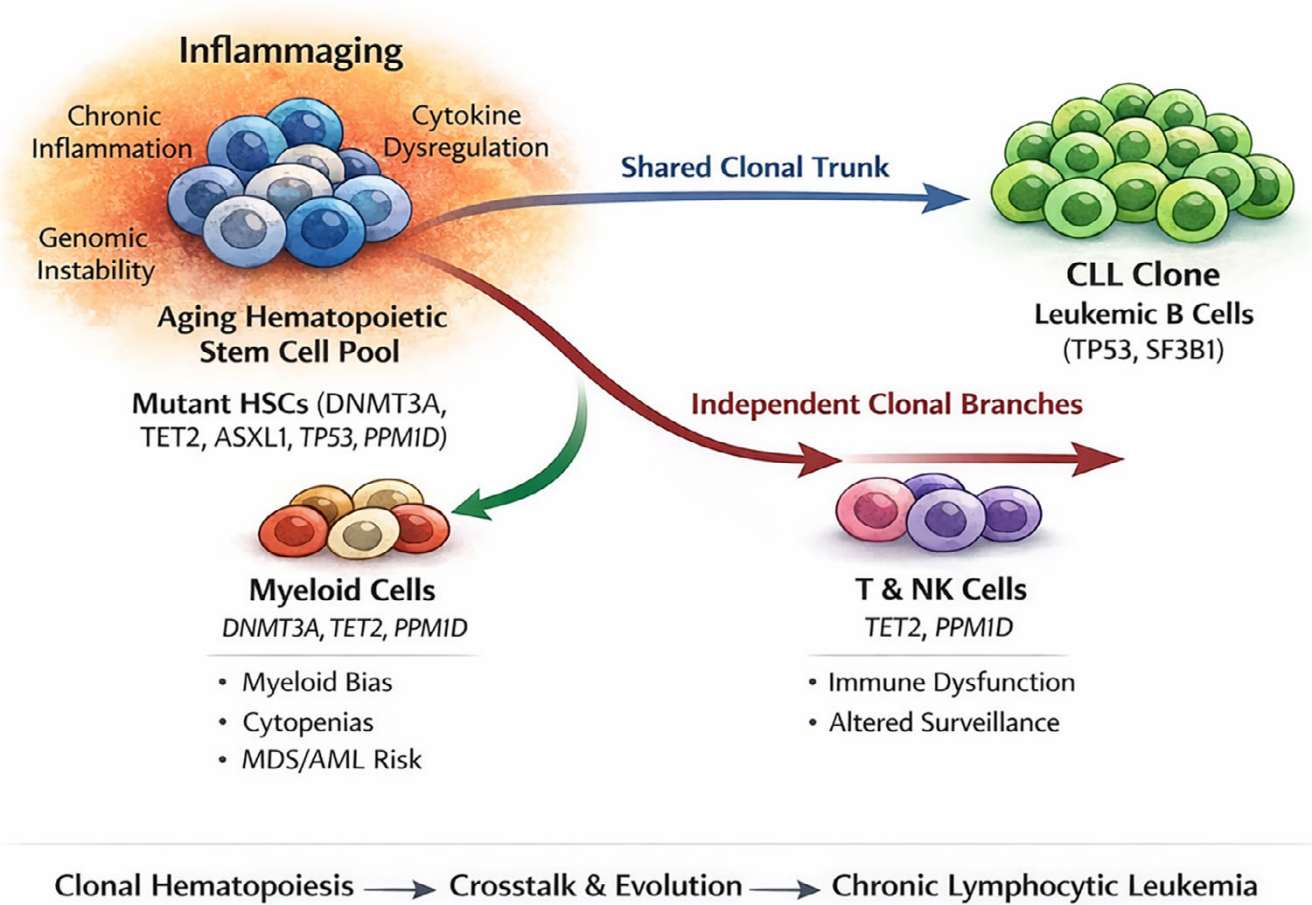
Clonal haematopoiesis (CH) and chronic lymphocytic leukaemia (CLL) within an inflammaging haematopoietic ecosystem. Ageing‐associated alterations in haematopoietic stem and progenitor cells (HSPCs) promote the emergence of CH, most commonly driven by mutations in DNMT3A, TET2, ASXL1, TP53 and PPM1D. In some patients, CH mutations arise in multipotent HSPCs and are subsequently captured within the CLL clone (shared clonal ‘trunk’), whereas in others CH remains confined to non‐B‐cell compartments, including myeloid and T/NK lineages (independent clonal branches). These processes occur within a background of chronic low‐grade inflammation (‘inflammaging’), shaping immune dysfunction, genomic instability and long‐term disease evolution.

Finally, CH and CLL should be viewed within the broader framework of age‐related chronic inflammation (inflammaging),^53^ which shapes clonal selection, comorbidities, treatment tolerance and the interconnected pathways linking ageing, CH and CLL development, as schematically summarised in Figure 1.

## 2 BIOLOGY OF CLONAL HAEMATOPOIESIS

CH is defined by the acquisition of somatic mutations or chromosomal alterations in HSPCs that confer a selective advantage and result in measurable clonal expansion in peripheral blood. In its most common definition—CHIP—CH involves mutations in genes linked to myeloid malignancies at a VAF ≥2%, without overt haematologic neoplasia or defined cytopenia.^13^, ^14^, ^16^ Related entities, such as age‐related clonal haematopoiesis (ARCH) and clonal cytopenia of undetermined significance, span a spectrum from benign mosaicism to pre‐malignant states.^54^, ^55^ CH also encompasses mosaic chromosomal alterations (mCAs), including copy‐neutral loss of heterozygosity and gains or losses of whole chromosomes, which may coexist with canonical CHIP and contribute to clonal dominance.^56^, ^57^

Sequencing of healthy individuals has identified recurrently mutated genes in CH, including epigenetic regulators (DNMT3A, TET2, ASXL1 and IDH1/2), DNA damage and cell cycle regulators (TP53, PPM1D and CHEK2), RNA splicing factors (SRSF2, SF3B1 and U2AF1) and signalling molecules (JAK2, CBL, NRAS and KRAS).^13^, ^15^, ^17^, ^23^ DNMT3A and TET2 mutations are most frequent, accounting for over half of cases.^58^, ^59^, ^60^ These lesions promote clonal fitness through altered epigenetic regulation, stress responses and inflammatory signalling. Spliceosomal mutations confer stem‐cell fitness advantages, particularly in older individuals or those with cytopenias.^17^, ^53^ PPM1D truncations, negative regulators of p53, expand under genotoxic stress and are enriched after cytotoxic therapy.^38^, ^39^, ^40^, ^60^ However, mechanistic pathways identified in experimental systems may not fully translate into human CLL biology and should be interpreted cautiously. JAK2 V617F increases thrombotic risk and predisposes to myeloproliferative neoplasms, while NRAS/KRAS mutations may remain indolent or contribute to myeloid/lymphoid neoplasia.^61^ In CLL, recurrent TP53 and SF3B1 mutations indicate that some lesions predate overt leukaemia and arise in multipotent HSPCs.^29^, ^62^, ^63^

Age is the strongest determinant of CH. CHIP is rare before age 40 years, but affects 10%–20% of individuals over 70 years, with higher prevalence detected by ultra‐deep sequencing.^13^, ^14^, ^15^, ^64^ This increase reflects cumulative mutational burden and age‐associated changes in haematopoietic fitness.

Ageing haematopoiesis is characterised by reduced HSC diversity, impaired lymphoid output, myeloid skewing, increased inflammatory signalling and diminished DNA repair capacity. Within this environment, HSPCs harbouring DNMT3A, TET2 or p53‐pathway mutations gain relative fitness. Experimental models show that Tet2‐deficient HSCs outcompete wild‐type cells under inflammatory stress,^24^, ^59^, ^65^, ^66^ although extrapolation to patient‐level outcomes remains cautious. In humans, inflammaging, oxidative stress and niche remodelling likely impose similar selective pressures.

Exogenous insults further shape CH evolution. Chemotherapy and radiotherapy selectively favour TP53‐ and PPM1D‐mutant clones, which often pre‐exist at low VAF and expand under treatment pressure rather than arising de novo.^38^, ^39^, ^40^, ^67^ This is particularly relevant in CLL, where prior cytotoxic exposure is common.

Longitudinal studies establish CH as a bona fide pre‐leukaemic state. In individuals who later develop AML, founding mutations in DNMT3A, TET2 and ASXL1, and related genes can be detected years before diagnosis and remain stable until acquisition of transforming lesions.^18^, ^68^, ^69^ CHIP confers a 10–13‐fold increased relative risk of haematologic malignancy, although absolute risk generally remains below 1% per year.^14^, ^16^, ^23^

The relationship between CH and lymphoid malignancies, including CLL, is more complex. In some patients, CLL driver mutations such as TP53 or SF3B1 are detectable in progenitor or myeloid compartments, indicating an HSC‐level origin.^28^, ^29^ In others, hallmark CLL lesions—such as immunoglobulin heavy chain variable region (IGHV) rearrangements or non‐coding deletions at 13q14—arise later during B‐cell differentiation and are lineage restricted.^70^, ^71^, ^72^ Accordingly, CH in CLL may represent a shared ancestral substrate, a parallel age‐related process or, in selected contexts, a contributor to therapy‐associated myeloid neoplasms. Emerging data suggest that DNMT3A‐mutant CH may be associated with adverse outcomes in some cohorts; however, these findings require validation in larger studies.^73^, ^74^, ^75^

CH has clinically relevant systemic effects independent of leukaemic transformation. CHIP is associated with increased risks of atherosclerotic cardiovascular disease, ischaemic stroke and all‐cause mortality.^20^, ^21^, ^22^, ^76^ Mechanistically, DNMT3A‐ and TET2‐mutant myeloid cells exhibit heightened inflammatory signalling, enhanced interleukin (IL)‐1β and IL‐6 production, and increased NLRP3 inflammasome activation, accelerating atherosclerosis in experimental models.^24^, ^25^, ^26^, ^27^ In CLL patients—often older and comorbid—CH‐associated inflammation may interact with treatment‐related toxicities, including atrial fibrillation and hypertension during BTKi therapy, although direct clinical evidence linking CH to ibrutinib‐associated cardiovascular events remains limited.^77^, ^78^

In summary, CH is characterised by age‐ and therapy‐modulated clonal selection with systemic consequences extending beyond leukaemia risk. In CLL, CH constitutes a dynamic haematopoietic background against which leukaemic clones and modern therapies interact, with implications for genomic interpretation and patient management.

## 3 INFLAMMAGING AND THE AGEING HAEMATOPOIETIC–IMMUNE SYSTEM

Ageing is associated with a chronic, sterile inflammatory state (‘inflammaging’), characterised by persistent elevations of IL‐6, tumour necrosis factor‐α, IL‐1 family cytokines, acutephase reactants and procoagulant factors, even in the absence of overt infection. This inflammatory milieu is a strong independent predictor of frailty, cardiovascular events, cognitive decline and all‐cause mortality, and represents a shared biological substrate for many age‐related disorders.^79^, ^80^

Inflammaging arises from multiple, overlapping mechanisms. Lifelong metabolic and genotoxic stress leads to the accumulation of damaged macromolecules and dysfunctional mitochondria, which act as danger‐associated molecular patterns and activate innate immune pathways, including Toll‐like receptors and the NLRP3 inflammasome, with tonic IL‐1β and IL‐18 production.^81^, ^82^ Age‐related increases in mucosal permeability and microbiome alterations permit low‐level translocation of microbial products, sustaining systemic immune activation.^83^, ^84^ Persistent viral infections, particularly cytomegalovirus, further reinforce chronic antigenic stimulation and cytokine release.^85^

Senescent cells also accumulate with age. Although transient senescence supports tissue repair, chronic persistence of senescent cells promotes inflammation through the senescence‐associated secretory phenotype, rich in cytokines, chemokines and proteases.^86^, ^87^ Clearance of senescent cells in animal models reduces multimorbidity and extends health span, underscoring their causal role in inflammaging.^88^, ^89^

These processes occur alongside immunosenescence. Adaptive immunity becomes contracted and oligoclonal, with reduced naïve Tcell output, expanded terminally differentiated clones, declining Bcell production and reduced repertoire diversity.^90^, ^91^ Innate immunity becomes dysregulated, exhibiting a pro‐inflammatory baseline state with impaired acute responses.^92^ Coagulation and complement systems similarly shift towards pro‐inflammatory and prothrombotic states, contributing to vascular and tissue injury.^93^

Inflammaging is not purely maladaptive. Pro‐inflammatory and stress‒response pathways are essential early in life but become detrimental when chronically active in old age, consistent with antagonistic pleiotropy.^90^ Centenarians often display elevated inflammatory markers yet remain functionally preserved, reflecting a balance between pro‐ and anti‐inflammatory mechanisms (‘anti‐inflammaging’).^94^, ^95^

Within this context, CH and CLL arise on a shared inflammaging background. Chronic inflammation, oxidative stress and antigenic stimulation reshape the haematopoietic stem cell compartment, favouring expansion of clones with mutations in epigenetic regulators, DNA damage response genes and splicing factors. CH drivers such as *TET2* and *DNMT3A* promote pro‐inflammatory myeloid differentiation and inflammasome activation, further amplifying systemic inflammation.^14^, ^82^ CH therefore both reflects and reinforces inflammaging. However, the directionality and magnitude of these interactions in CLL patients remain incompletely defined.

CLL arises within the aged haematopoietic system, particularly within the lymphoid compartment shaped by chronic antigenic and inflammatory exposure. Repeated antigen exposure and inflammatory cues favour expansion of B‐cell clones with stereotyped receptors, defective apoptosis and accumulating genetic lesions. Established CLL further perturbs immune homeostasis through infections, autoimmunity, hypogammaglobulinaemia and T‐cell exhaustion, increasing inflammatory load. Metabolic comorbidities—including obesity and type 2 diabetes—drive chronic systemic inflammation^96^, ^97^ and are associated with increased CH in population studies.^14^, ^98^ Inflammatory signalling enhances the fitness of DNMT3A‐ and TET2‐mutant clones in experimental models.^24^, ^25^ In CLL, where comorbidity burden affects outcomes,^99^ metabolic inflammation may promote CH expansion and influence disease course, although direct causal evidence in CLL‐specific cohorts remains limited.

Recognising inflammaging as the common soil for CH and CLL has important implications. Clinical risk in older CLL patients reflects not only leukaemia‐intrinsic genetics but also chronic inflammation and CH burden. Therapies that control leukaemia without addressing these factors may incompletely modify long‐term outcomes. Integrating inflammatory biomarkers and CH profiling into CLL studies may therefore improve risk stratification and inform strategies targeting both malignant and age‐related comorbid processes.

## 4 MOLECULAR LANDSCAPE OF CLL AND OVERLAP WITH CLONAL HAEMATOPOIESIS

The molecular landscape of CLL is defined by recurrent chromosomal abnormalities and somatic mutations that shape disease biology, prognosis and treatment response. Early prognostic stratification relied on FISH‐detected lesions, including deletion 13q14—often isolated and involving the DLEU2/miR‐15a/16‐1 locus—typically associated with indolent disease, and trisomy 12, deletion 11q (ATM) and deletion 17p (TP53), which correlate with aggressive behaviour and treatment resistance.^4^, ^5^ TP53 disruption remains one of the strongest adverse prognostic factors, predicting poor outcomes across therapies.^100^, ^101^

NGS expanded this framework by identifying recurrent mutations in NOTCH1, SF3B1, ATM, TP53, MYD88 and related pathways, defining biologically distinct CLL subsets.^6^, ^7^, ^8^, ^9^, ^102^ Despite a relatively low overall mutational burden, CLL consistently shows disruption of DNA damage response, RNA splicing, NOTCH and nuclear factor‐κB signalling, telomere maintenance and innate immune pathways. Deletion 13q14 exemplifies the pathogenic role of non‐coding alterations, as loss of miR‐15a/16‐1 deregulates BCL2 and cell‐cycle control,^70^, ^71^ and whole‐genome studies have revealed additional regulatory non‐coding mutations.^72^

CLL evolves through a multistep process. High‐count MBL shares many genomic features with overt CLL, including mutations in NOTCH1, SF3B1, ATM and POT1, but at lower variant allele frequencies and with fewer driver events, consistent with stepwise leukaemogenesis.^32^, ^33^, ^103^

Increasing evidence links CLL to haematopoietic stem cells from CLL patients can generate myeloid, T cell and leukaemic B‐cell progeny, indicating that some initiating lesions arise before B‐cell commitment.^28^ Ultra‐deep sequencing has detected SF3B1 and TP53 mutations in non‐B lineages at low allele frequencies, supporting a shared CH‐like precursor in a subset of patients.^29^

Studies using experimental models provide mechanistic support for the roles of key CH driver genes in haematopoiesis and clonal fitness. In mouse models, deletion of Dnmt3a or Tet2 specifically in haematopoietic cells increases stem cell self‐renewal and shifts differentiation towards myeloid lineages, resembling the clonal expansions observed in humans with CH.^24^, ^25^ Haematopoietic cells deficient in Tp53 or carrying truncating Ppm1d mutations demonstrate resistance to DNA damage, reflecting the selective expansion of pre‐existing clones during genotoxic therapy in CLL.^41^ Mutations in SF3B1 lead to widespread RNA splicing alterations, linking aberrant splicing to disrupted haematopoietic gene regulation.^104^ Similarly, loss of BAX impairs mitochondrial apoptosis, providing a survival advantage under therapeutic stress, with evidence that BAX mutations can expand in non‐B‐cell haematopoietic compartments during venetoclax therapy.^46^ Although these experimental findings offer valuable mechanistic insight, differences in species, microenvironmental context, and therapy exposure prevent direct extrapolation to human CLL. Consequently, they should be interpreted as supportive rather than definitive evidence for causal effects.

This overlap is most evident for TP53 and SF3B1. TP53 mutations define high‐risk CLL and are canonical CH lesions; TP53‐mutant clones may be positively selected by prior cytotoxic therapy, increasing the risk of therapy‐related myeloid neoplasms.^36^, ^38^, ^40^, ^63^, ^105^, ^106^

SF3B1 mutations occur in both poor‐risk CLL and CH/MDS, and their presence across multiple haematopoietic lineages supports an early, pre‐leukaemic origin in some cases, but unlike TP53, SF3B1 clones are not consistently expanded by cytotoxic therapy.^17^, ^29^ This dual role complicates genomic interpretation; however, careful interpretation is required because identical mutations may arise in CH, in the CLL clone, or both, and clinical consequences depend on the compartment and clonal burden.

Comprehensive genomic analyses suggest that CLL behaviour is determined by combinations of coding and non‐coding lesions layered onto an ageing, clonally distorted haematopoietic background.^8^, ^9^ Together, these data support a model in which CH may provide a permissive substrate for CLL development, with additional B‐cell‐specific events and microenvironmental selection driving overt leukaemia. Thus, CLL and CH represent partially overlapping evolutionary trajectories within an inflammaging haematopoietic ecosystem, a perspective that is essential for interpreting high‐sensitivity sequencing data and understanding therapy effects on both leukaemic and coexisting CH clones. These findings highlight that therapy may selectively expand CH clones, but causal links to CLL progression should be interpreted with caution.

In summary, CLL arises on a haematopoietic background that may already be clonally distorted by CH. Some mutations originate in multipotent progenitors, whereas others arise later in B‐cell differentiation. Understanding this layered architecture is crucial for interpreting high‐sensitivity sequencing data, distinguishing CH from leukaemic clones, and anticipating therapy‐driven clonal dynamics.

## 5 CLONAL HAEMATOPOIESIS IN PATIENTS WITH CLL

The recognition that CH is common in the ageing population has prompted investigation into its prevalence and relevance in CLL. Early evidence came from small, often single‐centre studies and was limited by major methodological challenges, notably the dominance of leukaemic B cells in peripheral blood and the overlap between canonical CLL driver genes and classical CH genes. Consequently, early reports likely underestimated both the frequency and complexity of CH in CLL.

Initial studies relied on targeted sequencing of unsorted peripheral blood in mixed cohorts, frequently including previously treated patients. Buscarlet et al. reported CH in a minority of CLL patients using panels focused on myeloid driver genes, with *DNMT3A* and *TET2* predominating, similarly to healthy controls.^47^ A subsequent analysis estimated CHIP (VAF ≥2%) in approximately 10%–20% of CLL patients, with higher prevalence after chemoimmunotherapy.^48^ While these studies established that CH is not rare in CLL, they could not reliably distinguish mutations arising in non‐B‐cell lineages from those confined to the leukaemic clone.

Later series, often enriched for patients exposed to fludarabine‐based regimens or alkylating agents, highlighted the interaction between therapy and CH. Voso et al. showed that patients who developed therapy‐related myeloid neoplasms (t‐MDS/AML) were more likely to harbor CH at baseline—particularly involving *TP53*, *PPM1D* and other DNA damage response genes—with post‐treatment clonal expansion consistent with therapy‐driven selection.^41^ Similar findings in broader oncology cohorts reinforced the view that CLL patients represent a population at increased risk for CH‐mediated late complications.^36^, ^38^, ^39^, ^40^

However, these studies shared substantial limitations. The main experimental and analytical approaches used to distinguish CH from CLL‐restricted mutations, together with their limitations, are summarised in Table 1. Most early reports analysed unsorted whole blood, where the abundance of CLL cells can distort VAF estimates of CH clones in myeloid or T‐cell compartments. Moreover, several genes central to CLL pathogenesis (*TP53*, *SF3B1* and *ATM*) are also recognised CH drivers, making clonal assignment difficult without lineage‐specific analyses or robust computational deconvolution.

**TABLE 1:** Clonal haematopoiesis (CH) in chronic lymphocytic leukaemia (CLL): prevalence, clinical associations and methodological considerations.

Aspect	Description/finding	Implications/clinical relevance	Methodological considerations/notes
Prevalence	CH detected in 40%–60% of CLL patients; CHIP (VAF ≥2%) ∼35%; higher after cytotoxic therapy	Indicates widespread pre‐leukaemic clonal architecture; may influence prognosis and therapy response	Detection depends on sequencing depth, panel design and lineage resolution
Gene drivers	Age‐related: DNMT3A, TET2, ASXL1; therapy‐related: PPM1D (chlorambucil), BAX/U2AF1 (venetoclax)	DNMT3A/TET2/ASXL1: cardiovascular risk, altered immunity; PPM1D/BAX: therapy‐selected clones	Include canonical CH genes and therapy‐associated genes in panels; consider lineage‐specific detection
Lineage distribution	CH may be confined to myeloid or T cells, or shared with the CLL clone (SF3B1, TP53)	Determines whether CH contributes to leukaemogenesis or is independent; may impact MRD interpretation	Use lineage‐sorted or single‐cell sequencing to resolve architecture
Clone size/VAF	Large clones (>10% VAF) and certain gene mutations (PPM1D, TP53) associated with adverse outcomes	Can inform risk‐adapted therapy selection and surveillance for therapy‐related myeloid neoplasms	High‐depth NGS required; report all detectable clones ≥2% VAF
Therapy impact	Venetoclax and BTKi can transiently select for BAX/U2AF1 clones; cytotoxic therapy increases prevalence	Monitoring is needed to anticipate haematologic toxicity, myeloid neoplasms and Richter transformation	Longitudinal sampling before, during and after therapy is recommended
Clinical outcomes	CH may influence cytopenias, infection susceptibility, progression‐free and overall survival (context dependent)	May guide choice between chemo‐free targeted regimens versus chemoimmunotherapy	Integrate CH genotype, VAF, lineage and therapy exposure in analyses
Sequencing method	Targeted NGS panels, WES/WGS, single‐cell or sorted sequencing	Sensitivity for low‐VAF clones and lineage attribution varies	High‐depth targeted NGS preferred; single‐cell or sorted approaches clarify clonal relationships
Sample source	Peripheral blood versus bone marrow; bulk versus lineage sorted	Bulk peripheral blood may dilute low‐VAF or lineage‐restricted clones	Consider CD19^−^/CD33^+^ separation or BM when feasible
Reporting/standardisation	Lack of uniform CH/CHIP definitions	Hinders cross‐study comparison	Follow consensus definitions: VAF ≥2%, absence of haematologic malignancy, gene restricted

Abbreviations: BM, bone marrow; BTK, Bruton tyrosine kinase; CHIP, clonal haematopoiesis of indeterminate potential; MRD, measurable residual disease; NGS, next‐generation sequencing; VAF, variant allele frequency; WES, whole‐exome sequencing; WGS, whole‐genome sequencing.

More refined studies addressed these issues through cell sorting and lineage‐resolved sequencing. Pleyer et al. demonstrated that mutations typically considered CLL drivers, including *SF3B1* and *TP53*, could be detected at low VAF in myeloid and T‐cell fractions, consistent with CH‐like pre‐leukaemic clones that precede and extend beyond the overt CLL clone.^29^ These observations indicate that CH may either provide an ancestral substrate for CLL development or evolve in parallel in independent HSPC‐derived lineages under the influence of age and therapy.

The most comprehensive evaluation of CH in CLL was provided by Al‐Sawaf et al. in 620 patients enrolled in the phase III CLL12 and CLL14 trials.^50^ Using error‐corrected targeted sequencing of 64 genes and a VAF threshold of  .5%, they showed that CH is present in the majority of CLL patients: 58.2% harboured at least one CH mutation and 35.5% met WHO criteria for CHIP. The mutational spectrum was dominated by *DNMT3A*, *TET2*, *TP53* and *ASXL1*, closely mirroring the general CHIP landscape but with higher prevalence. The principal CH genes and their biological and clinical correlates are summarised in Table 2. Additional cohorts beyond CLL12/CLL14 have reported significant CH prevalence in CLL patients. In a large retrospective cohort, myeloid CHIP was detected in ∼12% of untreated and ∼24% of treated patients, and in other realworld CLL cohorts DNMT3A, TET2 and ASXL1 mutations were frequently identified, with overall myeloid CH prevalence ranging from ∼24% to ∼47%.^51^, ^52^

**TABLE 2:** Biological features of clonal haematopoiesis (CH) relevant to chronic lymphocytic leukaemia (CLL).

Feature	Description	Representative genes	Biological consequences	Relevance to CLL
Definition	Expansion of haematopoietic clones carrying somatic mutations in individuals without overt haematologic malignancy	‒	Age‐dependent clonal skewing of haematopoiesis	Provides pre‐leukaemic background in which CLL arises
Age association	Prevalence increases with age; >50% in individuals >70 years with sensitive sequencing	DNMT3A, TET2, ASXL1	Altered self‐renewal and differentiation of HSPCs	Explains high CH prevalence at CLL diagnosis
Epigenetic dysregulation	Mutations affecting DNA methylation and chromatin remodelling	DNMT3A, TET2, ASXL1	Skewed lineage output, enhanced stem cell fitness, and inflammatory bias	Fosters a permissive haematopoietic and immune microenvironment
DNA damage response defects	Impaired sensing or repair of genotoxic stress	TP53, PPM1D, ATM	Resistance to apoptosis; survival under cytotoxic stress	Explains selective expansion after chemoimmunotherapy
Splicing alterations	Disruption of RNA splicing machinery	SF3B1, U2AF1, SRSF2	Aberrant transcript processing, genomic instability	Partial mutational overlap between CH and CLL clones
Inflammatory signalling	Enhanced pro‐inflammatory cytokine production	TET2, DNMT3A	Increased IL‐1β, IL‐6, TNF‐α; inflammaging	Contributes to immune dysfunction and CLL progression
Lineage restriction	CH mutations may be restricted to myeloid or lymphoid compartments	DNMT3A (pan‐lineage); TET2 (myeloid biased)	Differential clonal behaviour across haematopoietic lineages	Necessitates lineage‐sorted sequencing in CLL
Clonal size (VAF)	Clone size correlates with biological and clinical impact	High‐VAF TP53, PPM1D	Greater fitness advantage, increased pathogenicity	Large CH clones confer worse outcomes
Therapy responsiveness	CH clones respond differently to therapeutic pressures	BAX, U2AF1	Selection under targeted therapies	Impacts toxicity and long‐term risk under venetoclax
Systemic effects	CH influences non‐haematologic disease risk	DNMT3A, TET2	Cardiovascular disease, impaired immunity	Relevant for competing mortality in CLL

Abbreviations: HSPC, haematopoietic stem and progenitor cell; IL, interleukin; TNF‐α, tumour necrosis factor‐α; VAF, variant allele frequency.

A key strength of this study was the rigorous separation of CH from CLL‐restricted lesions. In CLL12, CH was assessed in CD19‐negative cells, while in CLL14 variant classification was integrated with minimal residual disease measurements at a time point of reduced leukaemic burden. This strategy directly addresses the methodological challenges outlined in Table 1 and likely explains the higher CH prevalence compared with earlier reports. Error‐corrected sequencing and lineage‐sorted approaches are increasingly available in specialised centres, although routine clinical implementation remains limited.

CH prevalence was higher in the older, more comorbid CLL14 population than in CLL12, consistent with age‐related expansion. Distinct therapy‐specific patterns emerged, with enrichment of *PPM1D* and *ASXL1* mutations after chlorambucil‐based therapy and *BAX* mutations under venetoclax‐based regimens—likely reflecting non‐B‐lineage CH under venetoclax pressure rather than direct CLL resistance—highlighting the impact of treatment‐driven clonal selection.

Overall, these data demonstrate that CH is highly prevalent and biologically relevant in CLL. Rather than arising in an otherwise normal haematopoietic background, CLL develops within an age‐ and treatment‐modified clonal haematopoietic ecosystem shaped by inflammaging, therapy‐driven selection and co‐evolving CH. This integrated view has important implications for immune dysfunction, treatment tolerance and long‐term outcomes in CLL patients.

## 6 IMPACT OF CLONAL HAEMATOPOIESIS ON CLL CLINICAL COURSE AND TREATMENT

CH influences CLL at multiple levels: disease biology and progression, therapy interaction, treatment‐related toxicities, therapy‐related myeloid neoplasms, Richter transformation (RT) and non‐CLL morbidity and mortality.

Whether CH directly accelerates progression is nuanced. Early retrospective series suggested that CHIP—particularly TP53 or splicing factor (SRSF2, U2AF1) mutations—might shorten time to first treatment and associate with adverse features, but these studies were limited by small sample sizes, heterogeneous therapies and incomplete adjustment for established risk factors.^48^, ^49^, ^51^, ^107^, ^108^

Prospective data from the CLL12 trial clarified this. Using error‐corrected sequencing of CD19‐negative cells, CH was prevalent even in early‐stage, asymptomatic CLL but did not independently predict progression once age and canonical risk markers were considered. However, large CH clones (VAF ≥10%) were associated with inferior overall survival in the observation arm, largely due to non‐CLL causes such as infections, cardiovascular events and secondary malignancies.^50^, ^109^ These findings indicate that in the ‘watch‐and‐wait’ phase, CH functions more as a marker of biologic ageing and vulnerability than as a direct driver of leukaemic progression, consistent with the concept of inflammaging.^20^, ^21^, ^110^

Multiple case–control studies across cancers, including CLL, have shown that nearly all tMDS/AML cases arise from pre‐existing CH, often years before overt disease, with TP53 and PPM1Dmutant clones selectively expanded by cytotoxic therapy.^36^, ^38^, ^39^, ^40^ In CLL, Voso et al. demonstrated that patients who develop tMDS/AML after chemoimmunotherapy almost always had detectable baseline CH, whereas matched CLL controls had lower frequency and smaller clone.^41^

**FIGURE 2:**
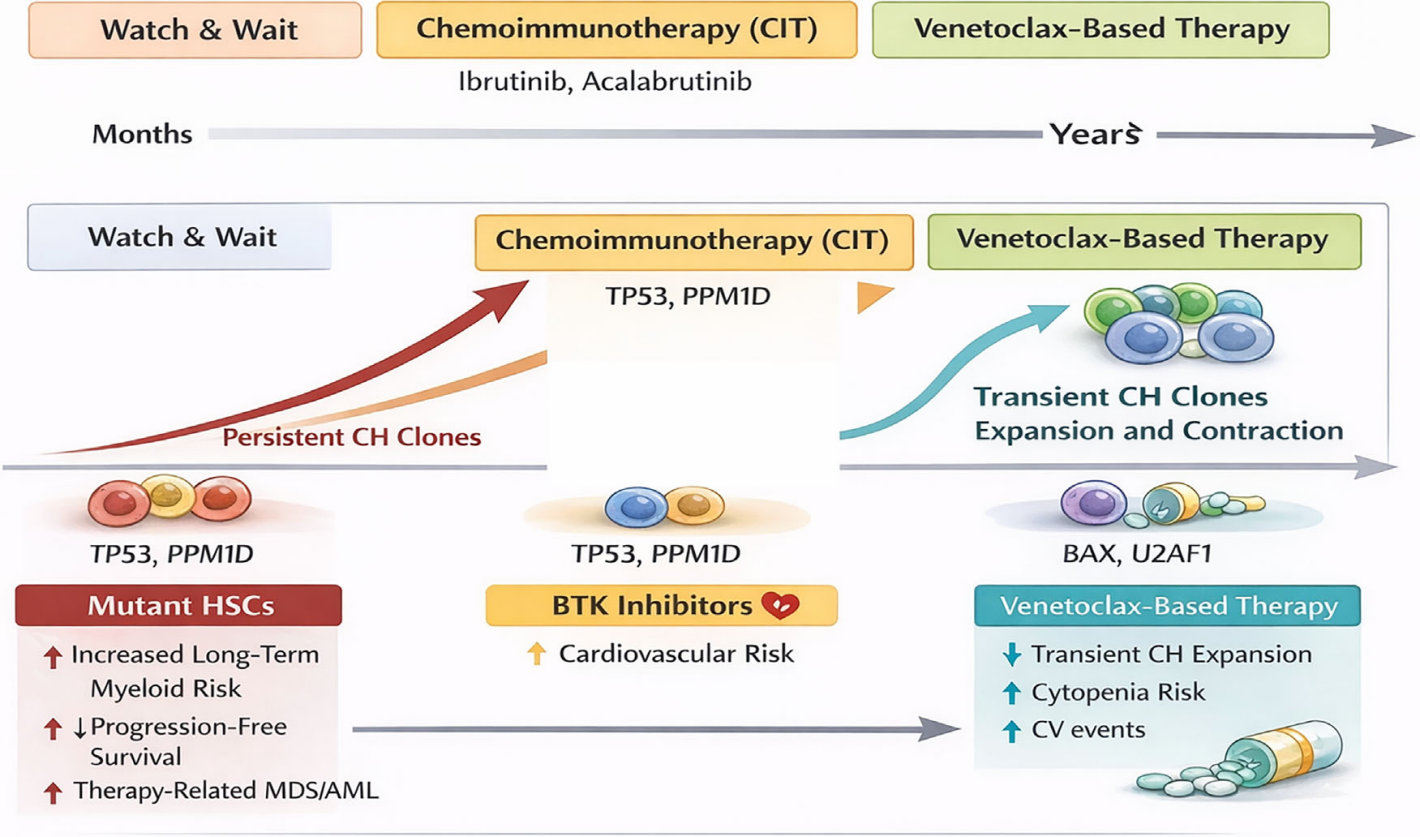
Therapy‐specific shaping of clonal haematopoiesis (CH) in chronic lymphocytic leukaemia (CLL). Chemoimmunotherapy exerts strong genotoxic pressure, selectively expanding CH clones with DNA damage response defects (e.g., TP53, PPM1D), thereby increasing the risk of therapy‐related myeloid neoplasms and inferior outcomes. Bruton tyrosine kinase (BTK) inhibitors exert minimal selective pressure on CH, while venetoclax‐based regimens transiently favour apoptosis‐resistant CH clones (e.g., BAX, U2AF1), which typically contract after treatment discontinuation. These therapy‐specific CH dynamics influence toxicity, late complications and long‐term risk.

Prospective data from CLL14 further support CH's impact under alkylator‐based therapy. Treatment‐naïve patients were randomised to Ven‐Obi or Clb‐Obi. In the Clb‐Obi arm, large CH clones (VAF ≥10%) predicted shorter progression‐free survival (PFS) after adjusting for age, IGHV status and del(17p), and PPM1D‐mutated CH predicted inferior overall survival. By contrast, in the Ven‐Obi arm, large CH clones did not affect outcomes.^44^, ^50^ These findings indicate that CH is clinically relevant under DNA‐damaging therapy, increasing risk of t‐MDS/AML and adverse CLL outcomes, particularly with TP53 or PPM1D mutations, and support avoiding genotoxic therapy when effective chemo‐free options exist. The differential effects of chemoimmunotherapy and targeted agents on CH dynamics and CLL outcomes are summarised in Figure 2.

Targeted agents, especially BTKis and venetoclax, largely mitigate CH's adverse effects. In CLL12, CH status and clone size did not affect PFS or overall survival in the ibrutinib arm, whereas in the placebo arm, large CH clones were associated with worse survival.^50^, ^111^ Ibrutinib does not appear to select for CH clones, although cardiovascular toxicity may intersect with CH‐associated risk.^77^, ^78^

Venetoclax can transiently select for CH clones with mutations in apoptotic regulators, such as BAX and U2AF1. In CLL14, these clones expanded during treatment but contracted after therapy ended, and their presence did not worsen PFS or overall survival.^45^, ^50^ BAX mutations likely reflect selective pressure on non‐B‐lineage CH rather than direct CLL resistance, consistent with findings in lineage‐sorted analyses. Functional studies confirmed that BAX loss confers a survival advantage under venetoclax. Fixed‐duration Ven‐Obi exerts transient selective pressure without compromising CLL control.

CH also influences treatment tolerability. CH‐positive patients in CLL12/CLL14 experienced higher rates of cytopenias, particularly neutropenia, during therapy, correlating with expansion of CH clones.^50^ Genes such as DNMT3A, TET2 and splicing factors can perturb haematopoiesis, reducing marrow reserve and increasing vulnerability to cytopenias under treatment.^16^

CH is associated in some studies with RT and secondary malignancies. Available data suggest that RT cases often co‐occur with CH, but evidence is limited to small cohorts and requires further validation with DNMT3A mutations contributing to the transformed clone either via expansion or low‐VAF infiltration.^50^, ^74^, ^75^ CH‐positive patients also had higher risk of secondary cancers, particularly haematologic malignancies driven by JAK2, DNMT3A, TET2 and ASXL1, consistent with non‐CLL populations.^110^, ^112^, ^113^ As CLL survival improves, CH‐mediated late effects will increasingly influence long‐term outcomes.

In summary, CH affects CLL multifactorially: it marks older patients at higher risk of non‐CLL mortality, increases susceptibility to therapy‐related myeloid neoplasms, modulates haematologic toxicity and contributes to RT and secondary malignancies. However, many findings remain preliminary and should be interpreted cautiously in clinical decision making. In the era of effective chemo‐free regimens, CH should inform treatment planning: minimising genotoxic therapy in high‐burden patients, favouring targeted approaches and ensuring long‐term surveillance for CH‐related complications.

## 7 INTEGRATING CLONAL HAEMATOPOIESIS INTO CLL MANAGEMENT AND RESEARCH

The recognition that CH is common and biologically meaningful in patients with CLL has substantial therapeutic and preventive implications, while raising numerous questions that warrant systematic investigation. These questions arise in the context of inflammaging, where chronic systemic inflammation, CH and CLL form an interconnected network rather than independent processes, influencing biology, prognosis, therapy and potential interventions.

A central issue is the lineage relationships between CH clones and the CLL clone. In some patients, CLL driver mutations such as SF3B1 and TP53 are detectable at low VAF in non‐B‐cell lineages, implying origin in HSCs before captured in the leukaemic B‐cell population.^28^, ^29^ In others, CH is restricted to myeloid or T/NK compartments, involving classical myeloid drivers (DNMT3A, TET2, ASXL1, PPM1D), without overlap with the CLL clone.^47^, ^50^

For practical clinical guidance, recommended approaches include analysing CD19‐negative fractions, interpreting VAF relative to leukaemic burden, correlating with measurable residual disease (MRD) when possible, and exercising caution when evaluating TP53 or SF3B1 variants, as these may originate from either CH or the leukaemic clone. Furthermore, low‐VAF DNMT3A, TET2 or PPM1D variants detected in routine peripheral blood NGS should be interpreted in the context of leukaemic burden, cytopenias and therapy history, as they may reflect CH rather than the leukaemic clone. These strategies provide a concise framework for distinguishing CH‐related mutations from CLL‐restricted lesions in research or high‐sensitivity sequencing settings.

Resolving these patterns at scale will require ultra‐deep, error‐corrected and lineage‐sorted and single‐cell sequencing with clonal phylogeny reconstruction, which remain primarily research tools and are not widely available for routine clinical practice. Most standard clinical NGS panels lack this resolution and can reliably detect variants only above ∼2%–5% VAF. These methods are essential for distinguishing CH from low‐burden leukaemic clones and for understanding CLL development, clarifying which CLL cases arise on a shared CH ‘trunk’, and whether they differ clinically from CLL developing on a polyclonal haematopoietic background, but their limited accessibility highlights the need to interpret high‐sensitivity data cautiously.

mCAs, which also occur in ageing haematopoiesis,^56^, ^57^ should be considered in future studies alongside CHIP to fully capture CH complexity.

**FIGURE 3:**
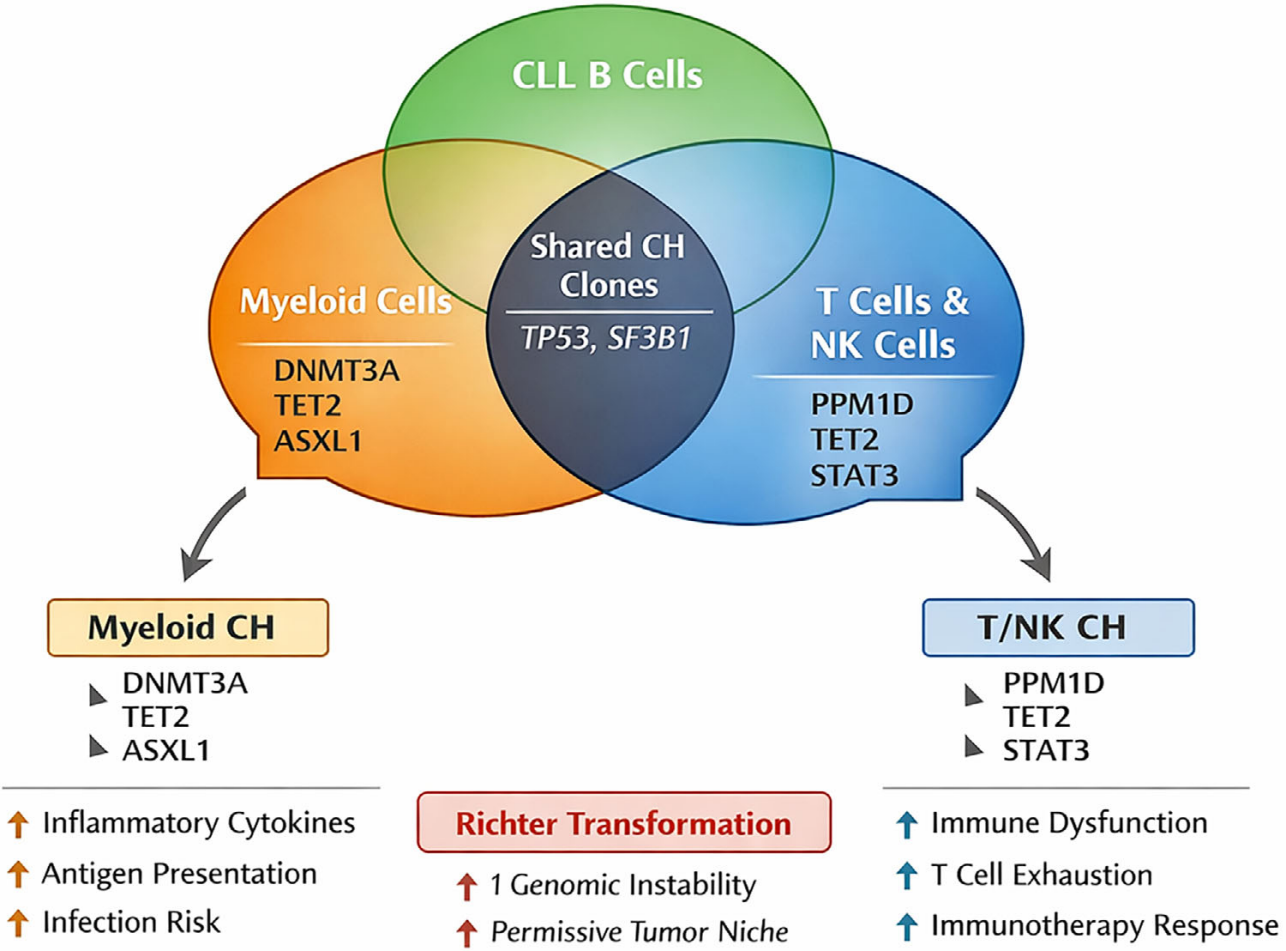
Lineage distribution of clonal haematopoiesis (CH) in chronic lymphocytic leukaemia (CLL) and effects on the tumour microenvironment. CH may be shared between CLL cells and non‐B‐cell lineages or remain restricted to myeloid or T/NK compartments. Myeloid‐restricted CH (e.g., DNMT3A, TET2, ASXL1) may drive chronic inflammation, impaired antigen presentation and infection susceptibility, whereas CH affecting T or NK cells may influence immune surveillance and responses to immunotherapy. These compartment‐specific effects contribute to disease progression, treatment tolerance and late complications such as Richter transformation.

The compartmental distribution of CH across CLL, myeloid and T/NK lineages, and its potential impact on the leukaemic microenvironment, is illustrated in Figure 3.

Closely related is how CH‐derived myeloid and T/NK cells influence the CLL microenvironment. CLL pathogenesis is heavily influenced by immune and stromal interactions, including nurse‐like cells, T cells, macrophages and mesenchymal stromal cells.^114^, ^115^ Myeloid cells carrying TET2 or DNMT3A mutations may exhibit altered inflammatory and phagocytic profiles, promoting chronic immune activation, infection susceptibility and impaired anti‐tumour responses, as suggested by murine models.^24^, ^25^ Similarly, CH‐affected T/NK cells may modulate immune surveillance and responses to CAR‐T cells or bispecific T‐cell engagers.^116^ Systematic phenotypic and functional characterisation of CH‐derived immune cells, including effects on immunotherapy outcomes, is a priority for future research.

Another key question concerns the therapy‐driven CH. Venetoclax‐associated expansion of BAX‐ and U2AF1‐mutated CH in CLL14^46^, ^50^ raises the issue of whether these clones confer long‐term risk of myeloid neoplasia. It remains unclear whether therapy‐selected CH patterns are unique to BCL2 inhibition or may occur with prolonged use of other targeted agents, such as next‐generation BTKis, PI3K inhibitors or bispecific antibodies. Current evidence suggests that fixed‐duration venetoclax limits sustained BAX‐mutant expansion, with no definitive increase in therapy‐related myeloid neoplasms, although ongoing follow‐up is required to confirm long‐term safety. Notably, BAX mutations likely represent non‐B‐lineage CH under venetoclax pressure rather than direct CLL resistance, highlighting the need to distinguish leukaemic from coexisting CH clones. Table 3 summarises CH dynamics across CLL therapies.

**TABLE 3:** Impact of clonal haematopoiesis (CH) across therapeutic contexts in chronic lymphocytic leukaemia (CLL).

Therapeutic setting	Predominant CH genes/patterns	Mechanism of CH selection or expansion	Clinical impact in CLL	Key evidence
Treatment‐naïve/watch‐and‐wait	Age‐related CH (DNMT3A, TET2, ASXL1); small clones	Physiologic age‐driven clonal drift; limited selective pressure	Generally neutral for TTFT; large clones may associate with inferior OS and non‐CLL mortality	^47^, ^98^
Chemoimmunotherapy (FCR, BR, chlorambucil based)	TP53, PPM1D, DDR‐related genes; expansion of pre‐existing CH	Genotoxic stress favours DNA‐damage‐resistant clones	Increased risk of prolonged cytopenias, t‐MDS/AML, inferior OS	^40^, ^41^, ^98^
BTK inhibitors (ibrutinib, acalabrutinib, zanubrutinib)	Persistence of age‐related CH; enrichment of non‐DNMT3A CH	Reduced genotoxic pressure; inflammatory and immune‐mediated selection	Minimal effect on PFS/OS; association with cardiovascular toxicity and atrial fibrillation	^71^, ^72^, ^98^
BCL2 inhibition (venetoclax‐based regimens)	Therapy‐selected BAX, U2AF1 CH; persistence of DNMT3A/TET2	Apoptotic pressure selects BAX‐deficient myeloid clones	Higher incidence of neutropenia; no clear adverse effect on PFS/OS	^46^, ^98^
Fixed‐duration Ven‐Obi	Transient CH expansion; limited clonal fixation	Time‐limited selective pressure	CH is largely neutral for survival outcomes	^98^
Continuous targeted therapy	Gradual CH clonal drift; possible accumulation of lesions	Prolonged low‐level selection over time	Long‐term consequences unknown; warrants surveillance	^46^
Post‐therapy/long‐term follow‐up	Persistence or expansion of high‐risk CH (TP53, splicing factors)	Therapy‐induced evolutionary bottlenecks	Increased risk of late myeloid neoplasms and non‐CLL mortality	^23^, ^38^

Abbreviations: BTK, Bruton tyrosine kinase; BR, bendamustine, rituximab; FCR, fludarabine, cyclophosphamide, rituximab; OS, overall survival; PFS, progression‐free survival; t‐MDS/AML, therapy‐related myelodysplastic syndrome/acute myeloid leukaemia; TTFT, time to first treatment; Ven‐Obi, venetoclax–obinutuzumab.

From a prognostic perspective, CH's role in routine CLL risk stratification remains unresolved. While CH increases hematologic malignancy and cardiovascular risk in the general population, its integration into CLL prognostic models, such as the CLL international prognostic index (CLL‐IPI), is not established.^117^ The incremental value of high‐risk CH genes or clone size thresholds (≥10% VAF) requires prospective evaluation in multivariable models, particularly in cohorts receiving frontline BTKis or venetoclax‐based regimens. CH prevalence in CLL does not appear substantially higher than in age‐matched controls, and this should be acknowledged when interpreting clinical significance.

Therapeutically, CH status could guide personalised interventions. CH‐positive patients, particularly with PPM1D or TP53 mutations, may preferentially receive chemo‐free regimens, while CH‐negative, genomically favourable patients may benefit from limited‐duration chemoimmunotherapy, analogous to MRD‐guided therapy adaptation in CLL.^45^, ^118^ However, these approaches remain exploratory, and current evidence does not support routine CH‐based therapy selection.

Preventive and interventional strategies remain exploratory. Routine CH screening is not recommended, although targeted testing may be justified in older patients, those receiving alkylator‐ or fludarabine‐based therapy, or individuals with cytopenias or strong familial predisposition.^16^, ^23^ Modulation of CH‐driven inflammation (e.g., IL‐1β or IL‐6 inhibition), targeted inhibition of specific CH drivers (IDH1/2, JAK2) before transformation, and lifestyle measures—diet, exercise, smoking cessation and aggressive cardiovascular risk management—to limit CH expansion and complications.

These strategies should be interpreted in the context of available NGS data, VAF thresholds and leukaemic burden, providing a practical framework for clinical interpretation of CH findings in CLL patients.

Interventions may include modulation of CH‐driven inflammation (e.g., IL‐1β or IL‐6 inhibition), targeted inhibitors of specific CH drivers (IDH1/2 and JAK2) before transformation, and lifestyle measures to limit CH expansion and complications.^2^, ^76^, ^113^ These strategies are investigational and should be applied cautiously within clinical studies.

Ethical and psychosocial considerations are also important. As sequencing sensitivity increases, incidental CH detection will become common, necessitating frameworks for counseling, risk communication and interdisciplinary management to balance awareness with potential anxiety or overtreatment.

In summary, CH in CLL is not merely a biological curiosity but a clinically actionable factor. Addressing the open questions—clonal architecture, microenvironmental effects, therapy‐driven dynamics, prognostic integration, CH‐guided treatment strategies, preventive approaches and ethical considerations—requires longitudinal, multi‐omic studies and CH‐stratified clinical trials. CH may also interact with chronic inflammatory conditions such as obesity, type 2 diabetes or metabolic syndrome, which are known to promote systemic inflammation and CH expansion, but evidence in CLL is currently limited. As evidence accumulates, CH is poised to become as integral to CLL management as IGHV mutation status, TP53 disruption and MRD assessment.

## 8 CONCLUSIONS

Over the past decade, CH has reshaped our understanding of ageing haematopoiesis and its clinical consequences. Large population studies show that CH—most commonly driven by DNMT3A, TET2, ASXL1, TP53 and PPM1D mutations—is frequent in older individuals and associated with increased risks of myeloid malignancies, cardiovascular disease and mortality, largely through altered inflammation and impaired DNA damage responses.^13^, ^14^, ^15^, ^16^, ^17^, ^20^, ^21^, ^23^

In CLL, CH adds an additional layer of biological and clinical complexity. The disease arises within an ageing, clonally distorted haematopoietic system shaped by age‐ and therapy‐influenced CH. Some mutations, notably TP53 and SF3B1, may originate in pre‐leukaemic haematopoietic stem cells and contribute to both CH and the CLL clone, whereas others—such as PPM1D or venetoclax‐selected BAX mutations—predominantly reflect therapy‐selected CH rather than therapy‐induced mutations.^38^, ^40^, ^41^, ^50^

Prospective trials now provide a coherent framework for interpreting the clinical relevance of CH in CLL. In CLL12 and CLL14, CH was present in ∼60% of patients, with ∼35% meeting CHIP criteria. Its impact was context‐dependent: large CH clones and PPM1D mutations adversely affected outcomes in untreated or chemoimmunotherapy‐treated patients, while CH had minimal influence on PFS or overall survival under ibrutinib or fixed‐duration Ven‐Obi.^50^ Additional cohorts beyond CLL12/CLL14 also report myeloid CHIP prevalence in 12%–24% of patients, confirming these findings.^48^

Beyond prognosis, CH influences treatment tolerance and late complications. CH‐positive patients experience more cytopenias—especially neutropenia during BCL2 inhibition—and have increased risks of therapy‐related myeloid neoplasms and RT, with DNMT3A‐ and TP53‐mutated clones frequently implicated. Myeloid‐restricted lesions such as TET2 or ASXL1 further increase risk, and transcriptomic studies suggest that CH may modulate immune dysfunction and responses to immunotherapy.^27^, ^50^, ^118^ However, the link between CH and RT remains preliminary and is primarily based on small cohorts; causal inference should be interpreted cautiously.

Therapy‐driven CH evolution has emerged as a recurring theme. Targeted therapies, including venetoclax and BTKis, can transiently select for CH clones such as BAX or U2AF1. While short‐term outcomes are generally unaffected, the long‐term consequences of therapy‐selected CH remain uncertain and warrant structured longitudinal follow‐up.^50^, ^119^, ^120^ These therapy‐selected CH clones likely reflect non‐B‐lineage haematopoietic populations rather than CLL resistance, emphasising the need for careful lineage‐resolved analysis.

Collectively, these data support a conceptual shift: CLL should be viewed not solely as a B‐cell malignancy, but as part of an age‐ and therapy‐modified, chronically inflamed haematopoietic ecosystem in which CH and the leukaemic clone co‐evolve. This interaction shapes disease behaviour, treatment toxicity, therapy‐related myeloid neoplasms, RT, cardiovascular events and late non‐CLL mortality. Recognition of CH—particularly in non‐B‐cell compartments—is therefore essential for accurate genomic interpretation, MRD assessment and comprehensive patient management. It is important to note that CH is common in older individuals, including those without CLL, and its prevalence in CLL does not appear substantially higher than that of age‐matched controls.^13^, ^14^, ^47^, ^52^, ^121^

Although CH profiling is not yet routine, near‐term clinical applications are emerging (Table 4). Baseline CH assessment may be considered in selected scenarios—for example, prior to genotoxic therapy, in patients with unexplained cytopenias, or in long‐term survivorship follow‐up to guide therapy selection towards chemo‐free regimens, anticipate haematologic toxicity under BCL2 inhibition, inform cardiovascular risk management, and tailor surveillance for therapy‐related myeloid neoplasms and RT. In transplantation, CH screening in older donors may also mitigate donor‐derived complications.^23^, ^122^ However, routine CH testing to guide initial therapy or cardiovascular risk management is not currently recommended, as evidence is preliminary.

**TABLE 4:** Clinical implications of clonal haematopoiesis (CH) in chronic lymphocytic leukaemia (CLL): risk stratification, monitoring and preventive strategies.

Clinical domain	CH features of relevance	Potential clinical implications	Current level of evidence	Key references
Baseline risk stratification	Presence of CH; large clone size (VAF ≥10%); high‐risk genes (TP53, PPM1D, splicing factors)	Identifies patients at higher risk of non‐CLL mortality, therapy‐related myeloid neoplasms, and treatment toxicity; may refine prognostic assessment beyond CLL‐IPI	Prospective evidence (CLL12/CLL14); not yet incorporated into guidelines	^47^, ^98^
Treatment selection	TP53‐ or PPM1D‐mutated CH; multiple CH lesions; large CH burden	Supports avoidance of genotoxic chemoimmunotherapy; favours chemo‐free targeted regimens (BTKi, Ven‐Obi)	Strong biologic rationale; supported by prospective trial data	^41^, ^98^
Prediction of haematologic toxicity	CH involving DNMT3A, TET2, ASXL1, splicing factors; CH expansion under therapy	Increased risk of cytopenias, particularly neutropenia under venetoclax; reduced marrow reserve	Prospective associations; requires validation for routine use	^51^, ^98^
Therapy‐related myeloid neoplasms (t‐MDS/AML)	Pre‐existing TP53, PPM1D, DDR‐related CH	Strongly increased risk after cytotoxic therapy; early identification enables surveillance and prevention	High‐level evidence across malignancies, including CLL	^38^, ^40^, ^41^
Richter transformation	DNMT3A‐ and TP53‐mutated CH; shared CH–CLL clonal architecture	Increased risk of RT; CH may contribute to the transformed clone or microenvironment	Emerging evidence from prospective cohorts	^68^, ^69^, ^98^
Cardiovascular risk	DNMT3A‐, TET2‐mutated CH	Increased risk of atherosclerosis and cardiovascular events; may interact with BTKi toxicity	Strong population‐level evidence; indirect CLL data	^20^, ^21^, ^71^
Long‐term surveillance	Persistent or expanding CH clones; therapy‐selected CH (e.g., BAX, U2AF1)	Guides monitoring for late myeloid neoplasms, secondary cancers and cytopenias	Long‐term impact still under investigation	^46^, ^98^
Preventive strategies	CH with inflammatory signatures; cardiovascular‐associated CH	Lifestyle modification, aggressive cardiovascular risk management, anti‐inflammatory approaches (investigational)	Conceptual and early clinical evidence	^70^
Ethical and counselling considerations	Incidental CH detection; low‐VAF clones	Need for structured patient counselling to balance awareness and anxiety; avoid overtreatment	Expert opinion	

Abbreviations: BTK, Bruton tyrosine kinase; CLL‐IPI, CLL international prognostic index; RT, Richter transformation; t‐MDS/AML, therapy‐related myelodysplastic syndrome/acute myeloid leukaemia; VAF, variant allele frequency; Ven‐Obi, venetoclax–obinutuzumab.

Looking forward, integrating CH into CLL care will require CH‐stratified prospective trials, longitudinal multi‐omic studies, and focused investigation of therapy‐driven CH evolution. As evidence accumulates, CH is poised to become a central component of risk‐adapted CLL management—alongside IGHV status, TP53 disruption and minimal residual disease—particularly in the context of inflammaging and an increasingly older patient population. Future studies should also examine interactions between CH and chronic inflammatory comorbidities such as obesity, type 2 diabetes or metabolic syndrome.

## AUTHOR CONTRIBUTIONS

All authors contributed to the manuscript and were involved in revisions and proofreading. All the authors approved the submitted version.

## CONFLICT OF INTEREST STATEMENT

The authors declare they have no conflicts of interest.

## FUNDING INFORMATION

The authors received no specific funding for this work.

## ETHICS STATEMENT

The study did not require ethical approval.

## Data Availability

Data sharing does not apply to this article as no datasets were generated or analysed during the current study.
